# Node Redeployment Algorithm Based on Stratified Connected Tree for Underwater Sensor Networks

**DOI:** 10.3390/s17010027

**Published:** 2016-12-24

**Authors:** Jun Liu, Peng Jiang, Feng Wu, Shanen Yu, Chunyue Song

**Affiliations:** 1 College of Automation, Hangzhou Dianzi University, Hangzhou 310018, China; liujun@163.com (J.L.); fengwu@hdu.edu.cn (F.W.); shanen_yu@hdu.edu.cn (S.Y.); 2 State Key Laboratory of Industrial Control Technology, Institute of Industrial Process Control, Zhejiang University, Hangzhou 310027, China; cysong@iipc.zju.edu.cn

**Keywords:** underwater sensor networks, node redeployment, self-examination and adjustment, stratified connected tree

## Abstract

During the underwater sensor networks (UWSNs) operation, node drift with water environment causes network topology changes. Periodic node location examination and adjustment are needed to maintain good network monitoring quality as long as possible. In this paper, a node redeployment algorithm based on stratified connected tree for UWSNs is proposed. At every network adjustment moment, self-examination and adjustment on node locations are performed firstly. If a node is outside the monitored space, it returns to the last location recorded in its memory along straight line. Later, the network topology is stratified into a connected tree that takes the sink node as the root node by broadcasting ready information level by level, which can improve the network connectivity rate. Finally, with synthetically considering network coverage and connectivity rates, and node movement distance, the sink node performs centralized optimization on locations of leaf nodes in the stratified connected tree. Simulation results show that the proposed redeployment algorithm can not only keep the number of nodes in the monitored space as much as possible and maintain good network coverage and connectivity rates during network operation, but also reduce node movement distance during node redeployment and prolong the network lifetime.

## 1. Introduction and Related Works

With rapid advances in technologies such as sensors, micro-electro-mechanical-systems (MEMS), wireless communication and embedded systems, wireless sensor networks (WSNs) have been widely used in applications like environment monitoring, military surveillance, industry or agriculture production, traffic control and heath care [1,2]. As application extension of conventional terrestrial WSNs, underwater sensor networks (UWSNs) are an kind of underwater monitoring system composed of nodes that are capable of information perception, data processing and communication, which also have such characteristics as using acoustic signal as communication medium, 3D network structure, limited and difficult-to-supplement node energy, node drift with water environment and relatively sparse node deployment density. Because of suitability for information acquisition in 3D underwater space, UWSNs have been widely used for seabed resources exploration, underwater pollution monitoring, and marine military [3,4,5]. Due to increasing attentions of many countries on marine resources, UWSNs have also become the research hotspot of current sensor network field. The UWSNs researches mainly involve node deployment [6,7,8], time synchronization [9,10], node localization [11,12], network route [13,14], energy balance and efficiency [15,16], etc. Among them, node deployment is not only closely related to network monitoring quality, but it is also the basis of other network protocol and algorithm designs [17].

There are many researches concerning UWSNs node deployment. According to different assumptions on node mobility, UWSNs node deployment algorithms can be classified into static, limited mobility and free mobility deployment [18]. The static deployment algorithm assumes that nodes are immovable and fixed at specific locations manually. Pompili et al. [19] proposed 2D and 3D UWSNs structures, and performed a deep mathematical analysis on node deployment on the basis of these two structures. Besides, they studied network robustness to node failures, and provided an estimate of the number of redundant sensors required. To realize full network coverage with the least nodes, Alam et al. [20] compared filling effect of truncated octahedron, cube, hexagonal prism and rhombic dodecahedron by using the idea of 3D Voronoi polyhedron tessellation. They found that truncated octahedron filling was the best strategy. Static node deployment algorithm can gain high network coverage and connectivity rates, and even realizes full network coverage and connectivity. However, it needed too large number of nodes and is not suitable for the sparse UWSNs deployment characteristic. Limited mobility node deployment algorithm assumes that nodes can only move vertically. Akkaya et al. [21] proposed a distributed self-organized node deployment algorithm, which reduced overlapped coverage by adopting the graph coloring idea. Nevertheless, this algorithm only emphasized on increasing network coverage rate and neglected network connectivity rate improvement. Hence, Senel et al. [22] put forward another distributed self-organized node deployment algorithm. It introduced in the connected dominating set (CDS) idea and maximized the network coverage rate under the premise of full network connectivity. Free mobility node deployment algorithm assumes that nodes can move toward all directions. Li et al. [23] proposed a 3D virtual forces deployment (TVFD) algorithm. It extended the 2D virtual forces deployment algorithm [24] to 3D space and was applicable to 3D underwater environment. Although it could improve network coverage and connectivity rates, it could not ensure full network connectivity (i.e., the network connectivity rate was 1). Besides, the network coverage rate could still be improved significantly. To realize effective coverage on events in the monitored underwater space, after proposing the fish-inspired node deployment algorithm [25], Xia et al. further put forward the similar particle swarm inspired node deployment (PSIND) algorithm [26]. This kind of algorithm made full use of behavioral features of fish or particle swarm, and was able to drive nodes to cover events as many as possible in the monitored underwater space.

As a research team which has been keeping much focus on WSNs, we have also proposed many node deployment algorithms for UWSNs in recent years [27,28,29,30,31]. A depth adjustment node self-deployment algorithm was proposed in reference [27], the main purpose of which was to improve the network connectivity rate achieved by the algorithm proposed in reference [21] and save network energy consumption caused by the algorithm proposed in reference [22]. An uneven cluster and radius-adjusting self-deployment algorithm was proposed in reference [28], the main purpose of which was to improve network reliability and balance energy consumption during network operation ignored by the algorithm proposed in reference [22]. These two algorithms belonged to limited mobility deployment, while the algorithm proposed in the following of this paper belonged to free mobility deployment, which also included our works proposed in references [29,30,31]. The work in reference [29] mainly focused on the node location determination and dispatch problem, the former was solved by the greedy iterative strategy where the connectivity nodes were used to improve the network connectivity rate until the full network connectivity was achieved and the latter was solved by the Kuhn-Munkres algorithm with the help of command nodes. The work in reference [30] proposed a node deployment algorithm based on CDS (DBCDS) algorithm to optimize the same network performances with that in reference [29]. However, different with that in reference [29], the network topology formed after the DBCDS algorithm operation has relationship with the initial random node scattering on the water surface. The node non-uniform deployment based on clustering (NNDBC) algorithm for UWSNs was proposed in reference [31], in which a high network connectivity rate was achieved by determining the heterogeneous communication ranges of nodes during node clustering, and the nodes with lower aggregate contribution degrees were used to substitute the dying nodes to decrease the total movement distance of nodes and prolong the network lifetime. The main difference between the NNDBC algorithm and our other works in references [27,28,29,30] as well as this paper was that the coverage targets in the NNDBC algorithm were isolated events, whose distributions were usually non-uniform in the monitored space.

In other words, those above researches did not fully consider node drift caused by water environment during network operation. Actually, if this is considered fully, the UWSNs node deployment problem can be converted into the UWSNs node redeployment problem. For study convenience, this paper defines the UWSNs node redeployment problem as periodic examination and adjustment on node locations with full consideration of the UWSNs characteristics (especially node drift with water environment during network operation). The main optimizing aim of this problem is to improve the network coverage and connectivity rates during network operation, reduce energy consumption during node redeployment as much as possible, and prolong the network lifetime as long as possible. Few researches on the UWSNs redeployment problem have been reported yet. Liu et al. [32] described the node drift with water environment during network operation with the 3D random drift model, and put forward the moving redundancy nodes redeployment (MRNR) algorithm. At every network adjustment moment, the sink node repeatedly requires the least important node, which makes the smallest contribution to the network coverage rate, to move toward the biggest coverage blind point. This could result in large network coverage rate improvement, but failed to keep nodes in the monitored underwater space effectively and did not consider how to improve the network connectivity during network operation. Moreover, this algorithm did not optimize the movement distance of the least important node during node redeployment. Due to limited node energy and huge energy consumption for node movement in underwater environment, nodes may die quickly upon running out of energy, which shortens the network lifetime.

There are only few UWSNs node redeployment algorithms, and they are difficult to improve or maintain the network coverage and connectivity rates as long as possible with the probably least energy consumption while taking full consideration of node drift with water environment. Therefore, this paper put forward a node redeployment based on stratified connected tree (NRBSCT) algorithm. In every network adjustment moment, self-examination and adjustment on node locations are performed firstly. If a node finds that it is outside the monitored space, it returned to the last location recorded in its memory along the straight line, which helps to maintain the network monitoring quality. Later, the network is stratified, and the whole network topology is changed into a connected tree that takes the sink node as the root node by broadcasting ready information level by level, which can improve the network connectivity during network operation. Finally, synthetically considering the network coverage and connectivity rates, together with the node movement distance, the sink node performs centralized optimization on locations of leaf nodes in the stratified connected tree. Simulation results show that the proposed redeployment algorithm can not only keep the number of nodes in the monitored space as much as possible, maintain good network coverage and connectivity rates during network operation, but also reduce node movement distance during node redeployment and prolong the network lifetime.

Compared with the existing related algorithms, the contributions of the proposed NRBSCT algorithm are as follows:

(1) It takes full consideration of node drift with water environment during network operation. At every network adjustment moment, nodes can keep themselves in the monitored space through self-examination and adjustment, which helps to maintain good network monitoring quality.
(2) At every network adjustment moment, the network is converted into a stratified connected tree through level-by-level stratifying, which can achieve full network connectivity and lower the network connectivity decrease speed during network operation.
(3) The sink node performs centralized optimization adjustment on the locations of leaf nodes in the stratified connected tree with synthetically considering the network coverage and connectivity rates, and node movement distance. This can not only maintain excellent network monitoring performances, but also reduce node movement distance during node redeployment, as well as prolong the network lifetime.

Table 1 is used to give a better description of the differences between the related research works and NRBSCT algorithm. The rest of the paper is organized as follows. In Section 2, the models, definitions and preliminaries involved in the NRBSCT algorithm are formally introduced. In Section 3, we elaborate the UWSNs node redeployment problem and corresponding NRBSCT algorithm. The simulation evaluation is provided in Section 4 with our conclusion and future work in Section 5.

## 2. Model, Definitions and Preliminaries

### 2.1. Models

#### 2.1.1. 3D Underwater Space Model

**Figure 1:**
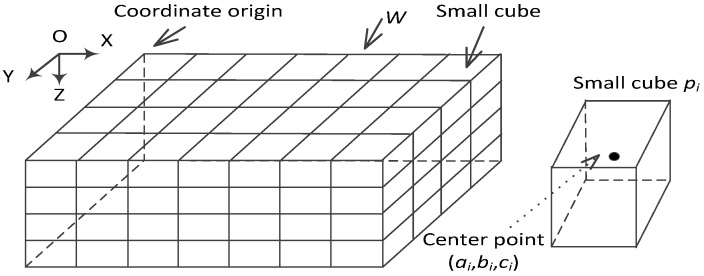
Coordinate system for UWSNs.

As is shown in Figure 1, the 3D underwater space is a large cube [33] divided into a number of small cubes whose side lengths are *w*. All the small cubes have selected their center points to represent themselves. The coordinates of cube *p_i_* is (*a_i,_b_i,_c_i_*).

#### 2.1.2. Node Energy Consumption Model

Considering that the energy consumption of nodes for sensing, processing, and receiving information is much smaller than for transmitting information and moving [34], only the latter is considered. The energy consumption for transmitting information is modeled based on the method mentioned in reference [35]. According to the related content in reference [35], the specific method to calculate the energy consumption for transmitting information can be elaborated as follows. Supposing that *d* denotes the geometrical transmitting distance of the information package, which includes the necessary information used in the network redeployment process, such as the ID number, location and left energy of the node, and the size of the information package is denoted as *M_b_. T_p_* denotes the transmitting time of the information package and *S_v_* is the transmission speed of the information package. On the one hand, we can calculate the transmitting time of the information package by the following formula:

(1) $$T_{p}=\frac{M_{b}}{S_{v}}$$

On the other hand, the relationship between the frequency *f* (KHZ) of the carrier acoustic signal and the water absorption coefficient *α*(*f*) (dB/m) is calculated by the following formula:

(2) $$\alpha \left(f\right)=0.11\frac{10^{-3}f^{2}}{1+f^{2}}+44\frac{10^{-3}f^{2}}{4100+f^{2}}+2.75\times 10^{-7}f^{2}+3\times 10^{-6}$$

When the transmitting distance of the information package is *d,* the energy attenuation is denoted as *A*(*d*) and can be calculated by the following formula:

(3) $$\{\begin{array}{l} A\left(d\right)=d^{\lambda }\times \beta^{d}\\ \beta =10^{\alpha \left(f\right)/10} \end{array}$$

where *λ* is the energy spreading factor (*λ* is 1 for cylindrical, 1.5 for practical, and 2 for spherical spreading). Therefore, the energy consumption for transmitting information can be denoted as *E_tx_*(*d*) and calculated by the following formula:

(4) $$E_{tx}\left(d\right)=P_{r}\times T_{p}\times A\left(d\right)$$

where *P_r_* denotes the power threshold for a node to receive the information package. Moreover, supposing that the number of information package transmitting times for node *s* in the network redeployment process is *t_n_*, and the communication range for node *s* is *R_t_*, the communication energy consumption *C_e_* can be obtained by the following formula:

(5) $$C_{e}=E_{tx}\left(R_{t}\right)*t_{n}$$

The movement energy consumption *M_e_* can be defined as the product of the movement distance *m_d_* and the energy consumption per movement distance *m_u_*, which can be also described as follows:

(6) $$M_{e}=m_{d}*m_{u}$$

#### 2.1.3. Node Sensing Model

The Boolean sensing model in reference [32] is adopted to describe node sensing. The function *f*(*p_i_*,*s_j_*) denotes whether cube point *p_i_* can be covered by node *s_j_*:

(7) $$f(p_{i},s_{j})=\{\begin{array}{ll} 1 & \left(\sqrt{{\left(x_{j}-a_{i}\right)}^{2}+{\left(y_{j}-b_{i}\right)}^{2}+{\left(z_{j}-c_{i}\right)}^{2}}\leq R_{s}\right)\\ 0 & \left(\sqrt{{\left(x_{j}-a_{i}\right)}^{2}+{\left(y_{j}-b_{i}\right)}^{2}+{\left(z_{j}-c_{i}\right)}^{2}}>R_{s}\right) \end{array}$$

where (*x_j_*,*y_j_*,*z_j_*) is the coordinate of node *s_j_*; (*a_i_*,*b_i_*,*c_i_*) is the coordinate of cube point *p_i_*, and *R_s_* is the sensing range of node *s_j_*. If the value of *f*(*p_i_*,*s_j_*) is 1, cube point *p_i_* is covered by node *s_j_*. Otherwise, cube point *p_i_* is not covered by node *s_j_*. Based on function *f*(*p_i_*,*s_j_*), coverage degree *k*(*p_i_*) of cube point *p_i_* can be defined as:

(8) $$k\left(p_{i}\right)=\sum_{j=1}^{n}f\left(p_{i},s_{j}\right)$$

where *n* denotes the total number of nodes in the network. Based on coverage degree *k*(*p_i_*), the function *f*_0_(*p_i_*) describes whether cube point *p_i_* is covered or not:

(9) $$f_{0}\left(p_{i}\right)=\{\begin{array}{ll} 1 & k\left(p_{i}\right)=0\\ 0 & k\left(p_{i}\right)\neq 0 \end{array}$$

If the value of *f*_0_(*p_i_*) is 1, cube point *p_i_* is not covered by any node.

#### 2.1.4. 3D Random Drift Model

The 3D random drift model mentioned in reference [32] is used to describe the node drift with water environment. The functions *rd*(*a*,*b*) and *rdi*(*a*,*b*) are used to produce the random real number and integer between *a* and *b*. These two functions play important role in simulating the random node drift with water environment, and *a* and *b* can be regarded as the parameters of these two functions. Whether a node drifts is expressed as follows:

(10) $$rd\left(0,1\right)<P_{e}$$

where *P_e_* is used to control the probability of node drift, and the higher *P_e_* means the larger node drift probability. If Equation (10) holds, the node drift model can be described as:

(11) $$\{\begin{array}{l} x_{i}=x_{i}+\lambda_{1}*rdi\left(0,m_{x}\right)*\left[2*\left(rd\left(0,1\right)<p_{dx}\right)-1\right]\\ y_{i}=y_{i}+\lambda_{1}*rdi\left(0,m_{y}\right)*\left[2*\left(rd\left(0,1\right)<p_{dy}\right)-1\right]\\ z_{i}=z_{i}+\lambda_{1}*rdi\left(0,m_{z}\right)*\left[2*\left(rd\left(0,1\right)<p_{dz}\right)-1\right] \end{array}$$

To explain the above model, the node drift in the *x* direction is taken as an example. *λ*_1_ and *m_x_* are the coefficients to determine the maximum drift distance along the *x* direction, and *P_dx_* is the coefficient to determine the drift probability along the *x* positive direction. The higher *P_dx_* means the larger node drift probability along the *x* positive direction. *rd*(0,1) < *P_dx_* is a Boolean expression whose value is 1 when it holds and 0 otherwise. The meanings of the similar coefficients in the *y* or *z* directions are like those in the *x* direction, and we omit the corresponding description about them for simplicity.

### 2.2. Definitions

#### 2.2.1. Network Coverage Rate

The network coverage rate *C_v_* can be defined as the ratio of *P_c_* and *P_t_*, where *P_c_* is the number of the cube points covered and *P_t_* is the total number of all the cube points. Therefore, *C_v_* can be calculated as follows:

(12) $$C_{v}=\frac{p_{c}}{p_{t}}$$

#### 2.2.2. Network Connectivity Rate

The network connectivity rate *C_n_* can be defined as the ratio of *n_c_* and *n*, where *n_c_* is the number of nodes that can communicate with the sink node through single-hop or multi-hop communication. *C_n_* can be calculated as follows:

(13) $$C_{n}=\frac{n_{c}}{n}$$

If the network connectivity rate is 1, the network achieves full network connectivity, and all the nodes can communicate with the sink node through single-hop or multi-hop communication.

#### 2.2.3. Network Adjustment Moment

The network operation time is expressed as *t* whose unit is denoted as round. If *t* ≠ 0 and is an integral multiple of the network adjustment cycle *T_r_*, this network operation time is also called the network adjust moment *T_ad_*, when the locations of nodes should be examined and adjusted to maintain or improve network monitoring quality.

#### 2.2.4. Energy Threshold

The energy of node *s_i_* is denoted as *E_i_*, and two kinds of energy threshold are defined in this paper. One is *E_d_* = *E_tx_*(*R_t_*) that is used to determine whether node *s_i_* is died. If *E_i_* is smaller than *E_d_*, we consider that node *s_i_* has almost depleted its energy and is hard to participate into the network monitoring, and we think that node *s_i_* is died. The other is *E_y_* = *E_tx_*(*R_t_*) × *T_r_* (*T_r_* is the network adjustment cycle) that judges whether a leaf node is strong. If the leaf node *s_i_* is a leaf node and its energy *E_i_* is smaller than *E_y_*, it is not strong enough and should be neglected during centralized optimization conducted by the sink node.

#### 2.2.5. Network Lifetime

The network lifetime is denoted as *L_f_*, which is one of the important criteria to evaluate the algorithm energy efficiency [36,37]. In this paper, the network lifetime is defined as the operating rounds where the network coverage rate *C_v_* satisfies the condition (i.e., *C_th_* ≤ *C_v_* ≤ 1), and *C_th_* is the coverage rate threshold. If the network coverage rate is smaller than *C_th_*, the network has difficulty in monitoring the underwater space, and the network lifetime is over.

### 2.3. Preliminaries

(1) Inspired by the related assumptions or descriptions in references [18,22], the sink node and all the other nodes can freely move in all directions with the help of related technologies such as AUVs [38] and their real-time locations can be known during network operation with the help of related localization technologies [39].
(2) Before the deployment, the destination location information of the sink node, i.e., the location information of the water surface center, has been stored in the memories of all the other nodes for them to gain information on the destination location of the sink node. Information on the 3D underwater space model and the number of nodes has also been stored in the memory of the sink node.
(3) The communication range of the sink node is *R_t_* and the sink node can be recharged, whereas the sensing capability of the sink node is neglected. All the other nodes are homogeneous, meaning these nodes have the same sensing range *R_s_*, same communication range *R_t_*, and initial energy *E_in_*. Furthermore, each of them has a unique *I_d_* number.

## 3. Problem and Algorithm Description

### 3.1. Problem Description

During the UWSNs network operation, as the 3D random drift model shows, node drift with water environment results in the network topology changes. Periodic examination and adjustment on node locations and node redeployment are needed to make the network maintain good monitoring performances as long as possible. There are few node redeployment algorithms considering node drift with water environment for UWSNs. Moreover, existing algorithms are difficult to improve the network coverage rate *C_v_* and connectivity rate *C_n_* at the cost of the probably least network energy consumption. For instance, as one of the typical node redeployment algorithms for UWSNs, the MRNR algorithm proposed that at every network adjustment moment *T_ad_*, the sink node repeatedly required the least important node, which made the smallest contribution to the network coverage rate, to move to the biggest coverage blind point through centralized optimization, aiming at achieving large network coverage rate improvement. However, this algorithm could not keep nodes in the monitored space effectively and did not consider how to improve the network connectivity rate. Moreover, it did not optimize the movement distance *m_d_* of the least important node during redeployment. Due to limited node energy and huge movement energy consumption *M_e_* in underwater environment, nodes die quickly upon running out of their energy (i.e., the left energy is smaller than *E_d_*), which shortens the network lifetime *L_f_*. Therefore, this paper proposes the NRBSCT algorithm. At every network adjustment moment, self-examination and adjustment on node locations are performed firstly. If a node finds it is outside the monitored space, it returns to the last location recorded in its memory along the straight line to maintain network monitoring quality. Later, the network is stratified into a connected tree that takes the sink node as the root node by broadcasting ready information level by level, which can improve the network connectivity rate during network operation. Finally, by synthetically considering the network coverage and connectivity rates, together with node movement distance, the sink node performs a centralized optimization on locations of leaf nodes in the stratified connected tree.

### 3.2. Algorithm Description

#### 3.2.1. Description of Initial Network Distribution

In this paper, two different initial network distributions are discussed. One initial network distribution is dented as D1 and the other is denoted as D2. The corresponding descriptions about them are given in the following respectively.

**Figure 2:**
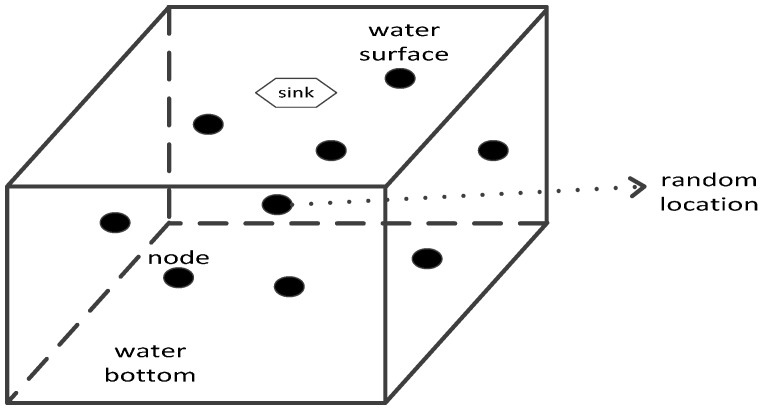
Initial network distribution D1.

Description about initial network distribution D1: As shown in Figure 2, the sink node is fixed at the center of the water surface. All the other sensing nodes are randomly scattered on the surface of the monitored 3D underwater space firstly, then randomly adjust their own depths. Therefore, this initial network distribution is exactly the random distribution and all the sensing nodes have random locations.

**Figure 3:**
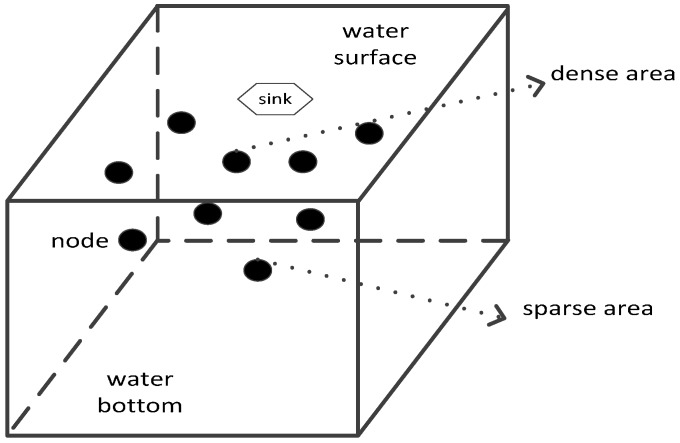
Initial network distribution D2.

Description about initial network distribution D2: As shown in Figure 3, the sink node is fixed at the center of the water surface. As to other sensing nodes, taking node *s_j_*(*x_j_*,*y_j_*,*z_j_*) for example and supposing that the coordinate of the sink node (i.e., the center of the water surface) is (*x_sink_*,*_ysink_*,0), the coordinate of node *s_j_* in the *x* direction follows the normal distribution whose mean is *x_sink_* and standard deviation is half of the *x* direction length of the monitored 3D underwater space. Similarly, the coordinate of node *s_j_* in the *y* direction follows the normal distribution whose mean is *y_sink_* and standard deviation is half of the *y* direction length of the monitored 3D underwater space. The depth of node *s_j_* (i.e., *z_j_*) can be calculated as follows:

(14) $$\{\begin{array}{l} d_{std1}=\sqrt{{\left(x_{j}-x_{sink}\right)}^{2}+{\left(y_{j}-y_{sink}\right)}^{2}}\\ d_{std2}=\sqrt{{x_{sink}}^{2}+{y_{sink}}^{2}}\\ z_{j}=\frac{d_{std1}}{d_{std2}}*z_{h} \end{array}$$

where *z_h_* means the depth of the monitored 3D underwater space. If the network follows the above distribution, the node distribution in the shallow area of the monitored 3D underwater space is denser than that in the deep area. Since the nodes closer to the sink node are usually burdened with heavier information packet forwarding task, this kind of distribution owns the effectiveness in balancing the network energy consumption.

#### 3.2.2. Algorithm Steps

Step 1: The network operation time *t* is initialized to be 0 round. The network adjustment cycle *T_r_* and node drift cycle *T_m_* are initialized to be two different non-zero constants, and the value of *T_r_* should be larger than that of *T_m_*.

Step 2: *t = t +* 1 round. Each node senses its own environment and transmits the sensing information to the sink node through single-hop or multi-hop communication. The relay node can compress the received sensing information from other nodes and its own sensing information into one information packet. Judge whether the value of *Rem*(*t,T_r_*) which means the remainder of dividing *t* by *T_r_* is 0 or not. If it is, the network operation time reaches the adjustment moment, and the algorithm turns to Step 3 for network topology adjustment; otherwise, the algorithm turns to Step 6.

Step 3: All nodes examine whether they are in the monitored space or not. If all of them are in the monitored space, the algorithm turns to Step 4 (when *t* = 0, all the nodes are in the monitored space); otherwise, the node outside the monitored space should return to the location recorded in its memory at the last network adjustment moment along the straight line.

**Figure 4:**
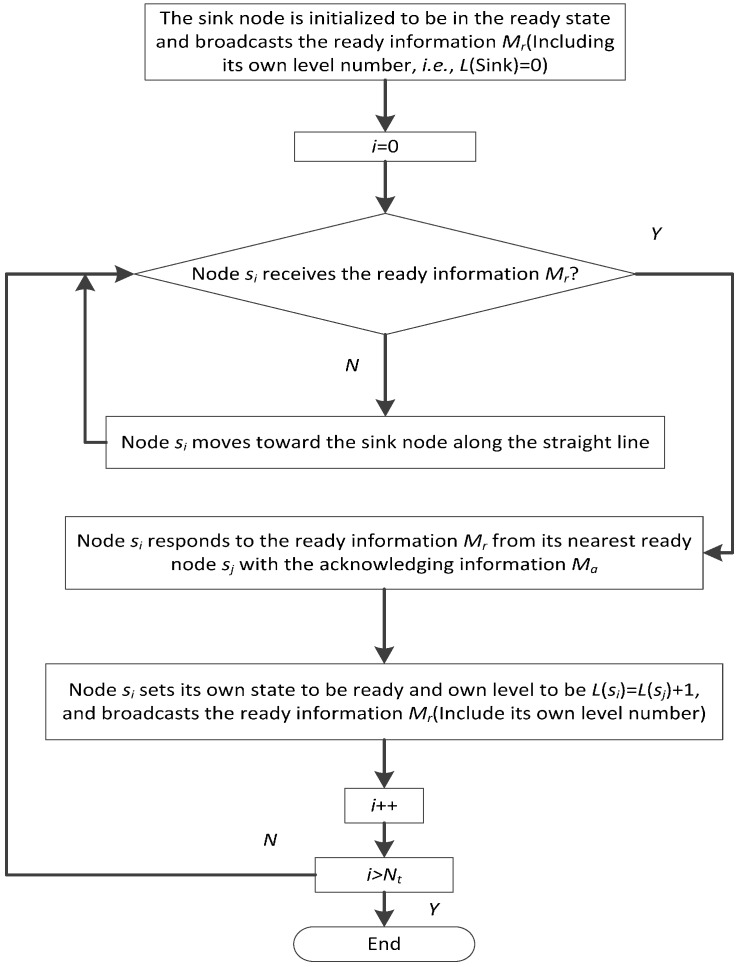
Flow chart description for network stratification.

**Figure 5:**
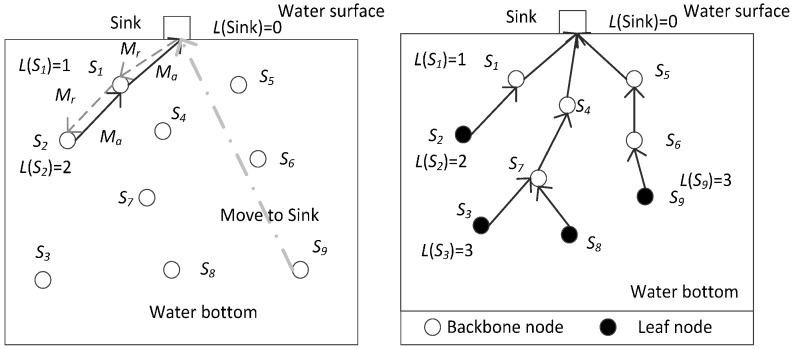
Network stratification process and result.

Step 4: The sink node set its state at the ready state and all the other nodes set their states at the non-ready states. If node *s_i_* has not received any ready information *M_r_*, it moves to the sink node along the straight line. During the movement, it opens its data receiving module to catch the ready information *M_r_* and stops moving upon the reception of *M_r_*. At the same time, the network is stratified level by level from the sink node. The corresponding description is shown in Figure 4. The process and result are shown in Figure 5 (a network comprised of 9 nodes is taken as the example). After finishing the network stratification, the whole network topology is changed into a stratified connected tree that takes the sink node as the root node, which helps to achieve full network connectivity. In the stratified connected tree, if node *s_i_* has received any acknowledging information *M_a_*, it has one or more child nodes and is considered as a backbone node; otherwise, node *s_i_* has no child node and is considered as a leaf node.

Step 5: After Step 4, the network achieves full network connectivity and the sink node can perform centralized adjustment. First, the sink node determines the set *C*_1_ comprising cube points in the 3D underwater space model, which are less than *R_t_* away (this is helpful to guarantee full network connectivity after centralized optimization) from the backbone nodes in the network. Next, it determines the set *C_y_* comprising the strong leaf nodes in the network. Then, new locations of leaf nodes in *C_y_* are calculated one by one according to the descending order of their residual energy. Taking leaf node *s_i_* for example, *p_i_* and *o_i_* are used to denote its probably new location and old location respectively. First, the sink node calculates its furthest movement distance *d_m_(i)* according to Equation (15):

(15) $$\{\begin{array}{c} d_{1}=\frac{E_{i}-E_{y}}{e_{mu}}\\ d_{2}=\frac{E_{i}\times r_{g}}{e_{mu}}\\ d_{m}(i)=min\left\{d_{1},d_{2}\right\} \end{array}$$

where *d*_1_ ensures that *s_i_* is still a strong leaf node after reaching the probably new location and *d*_2_ ensures that *s_i_* will not consume excessive energy during the movement (*r_g_* is the control coefficient). Both of them limit the movement energy consumption of the leaf node from different perspectives (*d*_1_ is from the left energy perspective and *d*_2_ is from the consumed energy perspective), therefore, the node movement distance is reduced. Second, the sink node determines set *C*_2_ comprising cube points in the sphere *Q_i_*, whose center is the old location (i.e., *o_i_*) of node *s_i_* and radius is *d_m_*(*i*). The set *C_d_* = *C*_1_*∩C*_2_ is calculated. Next, the sink node considers cube points in *C_d_* one by one. Taking cube point *p_i_* for example, the network coverage rate is *C_v_*(*o_i_*) when node *s_i_* is at its old location *o_i_*, and would be *C_v_*(*p_i_*) if node *s_i_* moved to its probably new location *p_i_*. The distance between *o_i_* and *p_i_* is denoted as *d*(*o_i_,p_i_*). The target cube point *p_d_*(*s_i_*) (i.e., final destination location of node *s_i_*) is calculated according to Equation (16). The sink node returns the result information to node *s_i_*, and node *s_i_* moves to *p_d_*(*s_i_*) along the straight line. Then, the sink node considers the next strong leaf node until all the strong leaf nodes have been studied. After this, all the nodes record the real-time locations into their own memories.

(16) $$\{\begin{array}{c} p_{d}(s_{i})=\underset{p_{i}\in C_{d}}{\max}\left\{\frac{\Delta C_{v}(o_{i},p_{i})}{d(o_{i},p_{i})}\right\}\\ \Delta C_{v}(o_{i},p_{i})=C_{v}(p_{i})-C_{v}\left(o_{i}\right)>0 \end{array}$$

Step 6: The fact that whether *Rem*(*t*,*T_m_*) (remainder of dividing *t* by *T_m_*) is 0 or not determines whether the node drift with water environment occurs in the current network operation time. If the value of *Rem*(*t*,*T_m_*) is 0, all the nodes drift according to the 3D random drift model; otherwise, the algorithm turns to Step 7.

Step 7: The sink node calculates the network coverage rate and judges whether the network reaches the network lifetime or not. If it is, the algorithm ends; otherwise, the algorithm turns to Step 2.

#### 3.2.3. Detailed Description for Algorithm Important Parts

To give a better understanding about the proposed algorithm, the following important parts of the NRBSCT algorithm will be elaborated in detail and with examples. Supposing that the scenery is the same with that described in the following simulation setup, and the network adjustment cycle *T_r_* and node drift cycle *T_m_* are initialized to be 50 rounds and 5 rounds respectively. The network operation time just comes to 50 rounds, i.e., the first adjustment moment. At this time, the algorithm will turn to Step 3 which is mentioned in the previous part for network topology adjustment. We will take the scenery at this time as an example to describe for simplicity.

##### Self-Examination and Adjustment of Nodes Outside the Monitored Space

Since the network operation time is just 50 rounds, actually all the nodes have drifted according to the 3D random drift model 9 times, which occurred at Rounds 5, 10, 15, 20, 25, 30, 35, 40, and 45. It is possible that some nodes may drift outside the monitored space. Taking node *s_e_* as the example, as is shown in Table 2, it gradually drifted outside the monitored space, so it returns to the location recorded in its memory at the last network adjustment moment. Since the network operation time is just the first adjustment moment, the last network adjustment moment means the operation starting time (i.e., when *t* = 0). Therefore, node *s_i_* will move from (125, 43, 21) to (115, 37, 16), which makes itself come back into the monitored space to help maintain good network monitoring quality.

##### Network Connectivity Rate Improvement

After Step 3, all the nodes have been into the monitored space. However, because of the initial random deployment and previous drift caused by the water environment, some nodes fail to communicate with the sink node through single-hop or multi-hop communication, which means that the network cannot still achieve full network connectivity. Therefore, measures should be taken to improve the network connectivity, and it is better to achieve full network connectivity if possible. Taking the scenery shown in Figure 5 as an example, 9 nodes in the network finally form a stratified connected tree whose root node is the sink node with the method described in Figure 4 in Step 4. This is one aspect of improve the network connectivity rate for the NRBSCT algorithm. As to the other aspect, in the centralized adjustment performed by the sink node in Step 5, the target cube point *p_d_*(*s_i_*) (i.e., final destination location of strong leaf node *s_i_*) belong to the set *C_d_* = *C*_1_*∩C*_2_, which means it also belongs to the set *C*_1_ and is less than *R_t_* away from the backbone nodes in the network, which is helpful to guarantee full network connectivity after centralized optimization. The scenery shown in Figure 5 can also be the example. Figure 5 shows that after the network stratification, nodes *s*_2_, *s*_3_, *s*_8_ and *s*_9_ are the leaf nodes. Supposing that their left energy are distributed as Table 3 shows (the simulation value at the 50 round time is much bigger than the supposition value in Table 3), since the energy threshold *E_y_* = *E_tx_*(*R_t_*) × *T_r_* is 65 J in the currently supposed simulation scenery, nodes *s*_2_ and *s*_8_ can be treated as the strong leaf nodes. Therefore, based on Equations (15) and (16), the sink node firstly determine the final destination location for node *s*_2_, and then for node *s*_8_.

##### Node Movement Distance Limit

**Figure 6:**
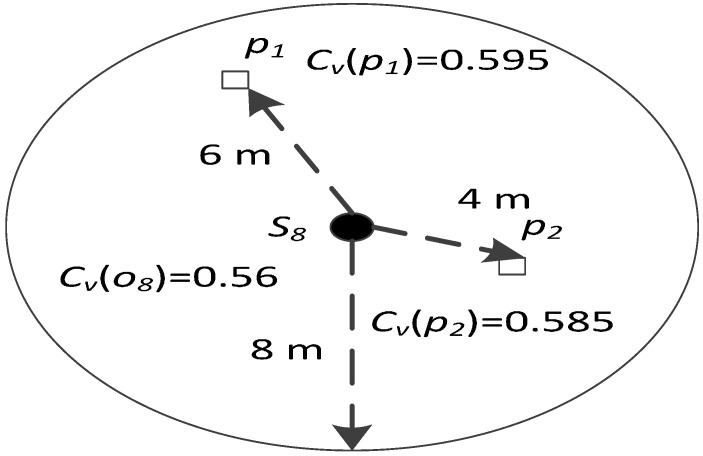
Illustration for final destination location chosen.

During the calculation of the final destination location for the strong leaf node, it is necessary to consider how to limit the node movement distance, since the movement energy consumption is huge and node energy is limited and difficult-to-supplement in the water environment. Therefore, in Equations (15) and (16), measures are taken to limit the node movement distance. Taking node *s*_8_ as the example, in Equation (15), the calculation of *d*_1_ (value is 8 m) limits the movement distance by ensuring that node *s*_8_ is still strong after the current movement; while the calculation of *d*_2_ (value is 10 m) limits the movement distance by ensuring that node *s*_8_ will not consume excessive energy during the current movement. By using Equation (16), the node movement distance is also limited when requiring the node to move toward its final destination location to improve the network coverage rate. As is shown in Figure 6, point *p*_1_ and *p*_2_ belong to the set *C_d_* = *C*_1_*∩C*_2_*.* If node *s*_8_ chooses *p*_1_ as its final destination location, it should move 6 m to gain 0.035 network coverage rate improvement; however, if node *s*_8_ chooses *p*_2_ as its final destination location, it should move 4 m to gain 0.025 network coverage rate improvement. Since moving toward *p*_2_ can gain more network coverage rate improvement per movement distance, node *s*_8_ will prefer to choose *p*_2_ rather than *p*_1_.

##### Overall Summarization for Main Mathematical Symbols

Since there are a large number of mathematical symbols, to give a better description of these symbols, Table 4 includes main mathematical symbols used.

#### 3.2.4. Algorithm Analysis

For the 3D underwater space model, whether a small cube is covered or not depends on the covering state of its center point. Therefore, for the NRBSCT algorithm, when the sink node performs centralized optimization to adjust the locations of leaf nodes, the cube resolution *w* affects the calculating accuracy and time complexity of centralized optimization. If *w* is small, the calculating accuracy and time complexity is high. For example, when the sink node calculates the final destination location for node *s_i_*, it has to calculate *C_v_*(*o_i_*) and *C_v_*(*p_i_*), and the time complexities for these two calculations are the same, i.e., *O*(*P_t_* *×* *n*). The smaller *w* means the larger *P_t_*, and then results in the higher time complexity.

## 4. Simulation Evaluation

### 4.1. Algorithm Comparison and Evaluation Metrics

Though the MRNR algorithm seems to be a little old, it seems the only one that can be found to solve the node redeployment problem researched in this paper as far as we know after our deep and extensive literature study; thus, to evaluate the proposed NRBSCT algorithm reasonably, the MRNR algorithm is chosen for comparison. The performances of MRNR and NRBSCT algorithms are compared from the following metrics: the number of nodes in the monitored 3D underwater space, network coverage rate, network connectivity rate, and network lifetime.

Compared with the MRNR algorithm, the NRBSCT algorithm has some advantages, as follows:

(1) The MRNR algorithm does not propose effective measures to prevent nodes from drifting out of the monitored space because of water environment. However, the NRBSCT algorithm can make nodes outside the monitored space return back through self-examination and adjustment on node locations, which is goodfor maintaining good network monitoring quality.
(2) The MRNR algorithm only considers how to improve the network coverage rate, but ignores the network connectivity rate improvement. However, at the network adjustment moment, the NRBSCT algorithm can firstly establish a stratified connected tree that takes the sink node as the root node, and then ensure that in the centralized optimization conducted by the sink node, the final destination location of node *s_i_* will not be *R_t_* away from the backbone nodes, which achieves full network connectivity and also helps to maintain a relatively high network connectivity during network operation.
(3) The MRNR algorithm does not consider how to shorten the movement distance when requiring the least important node to move toward the biggest coverage blind point to improve the network coverage rate. However, the centralized optimization conducted by the sink node in the NRBSCT algorithm considers limiting node movement distance from the left and consumed energy perspectives, which can shorten the total movement distance of nodes during redeployment and prolong the network lifetime.

### 4.2. Simulation Scenario and Parameter Settings

Matlab software is used to simulate the algorithms. The final results shown in the following figures are the averages of 50 times to eliminate the effect of simulation randomness. The length and width in the horizontal direction of simulative monitored 3D underwater space are the same, i.e., 120 m, whereas the depth of space is 60 m. The cube resolution *w* is 5 m. In the 3D random drift model, node maximum drift distances along the *x*, *y* and *z* directions are controlled by setting *m_x_* = *m_y_* = *m_z_* = 1 and *λ*_1_ = 0.8. Node drift probability *P_e_* is set to be 0.3, and drift probabilities along the positive directions of *x*, *y* and *z* axes are controlled by setting *P_dx_* = *P_dy_* = *P_dz_* = 0.5. In the NRBSCT algorithm, the value of *r_g_* is 0.2. Other main parameter settings of the MRNR and NRBSCT algorithms are enumerated in Table 5.

### 4.3. Simulation Results and Analysis

**Figure 7:**
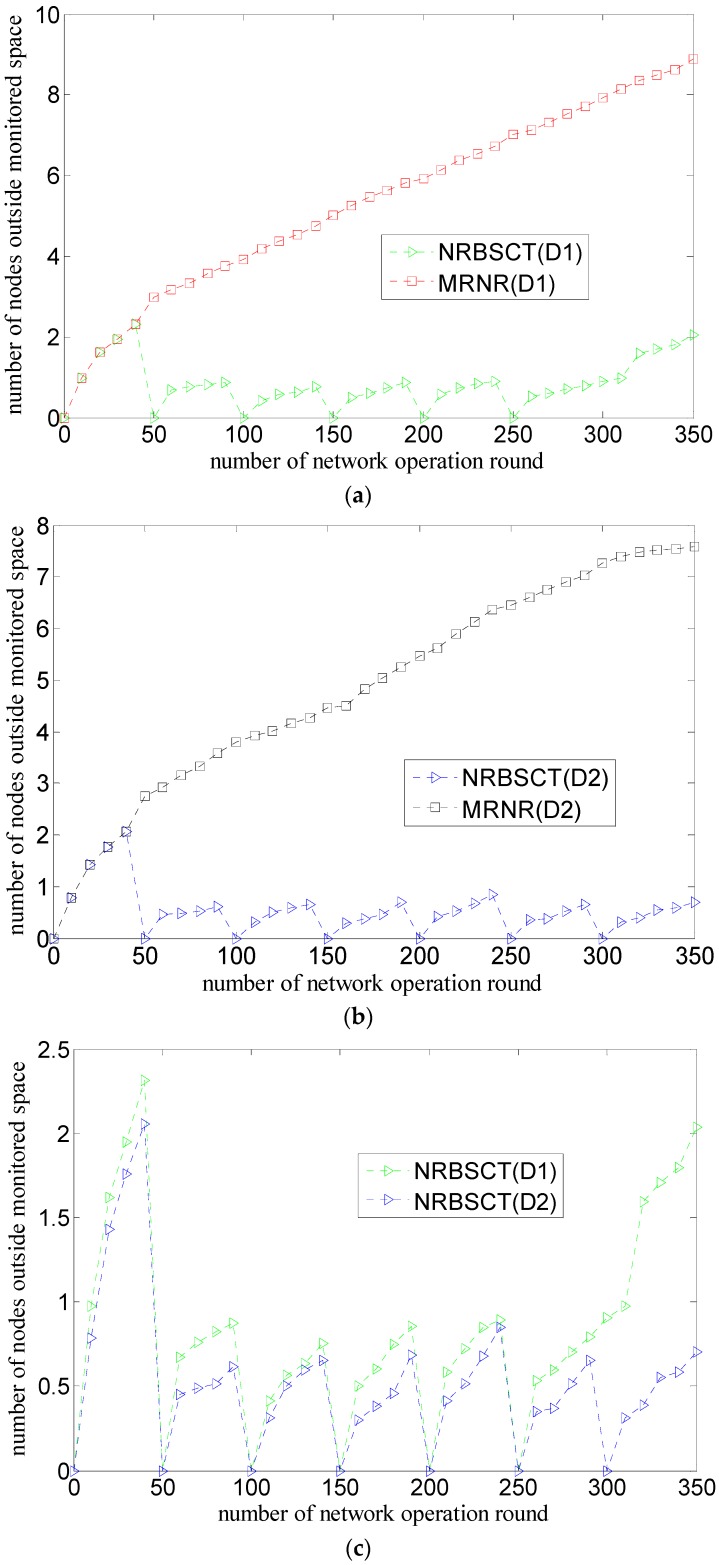

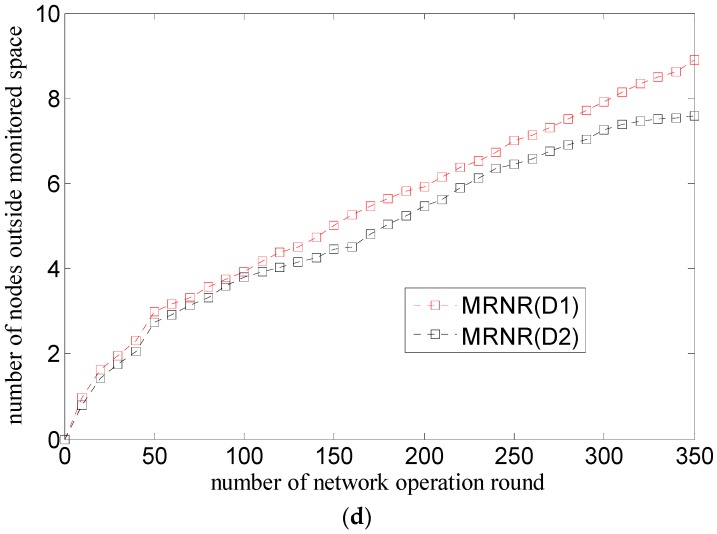
Comparison of number of nodes outside monitored space during network operation: (**a**) different algorithms and same network distribution (D1); (**b**) different algorithms and same network distribution (D2); (**c**) different network distribution and same algorithm (NRBSCT); and (**d**) different network distribution and same algorithm (MRNR).

Figure 7 shows the comparison of the relationship between the number of nodes outside the monitored space and the round of network operation considering both the initial network distribution (D1 or D2) and the network redeployment algorithm (MRNR or NRBSCT), where the number of nodes is 30.

As shown in Figure 7a,b, node may drift with water environment during network operation. For the MRNR algorithm, more and more nodes drift outside the monitored space during network operation; however, for the NRBSCT algorithm, the number of nodes outside the monitored space does not increase too much, moreover, the NRBSCT algorithm can almost keep all the nodes in the monitored space when the network operation time (not exceeding the network lifetime) is just the network adjustment moment. The reason is that the MRNR algorithm does not propose any methods to prevent nodes from drifting outside the monitored space because of water environment; however, the NRBSCT algorithm can drive nodes outside the monitored space to move back into the monitored space through self-examination and adjustment on node locations, which is significant to help the network maintain better monitoring quality.

As shown in Figure 7c,d, for both the MRNR algorithm and the NRBSCT algorithm, compared with the initial network distribution D1, since there are more nodes distributed in the area around the sink node and less nodes distributed in the area near the boundary of the monitored water space for the initial network distribution D2, less nodes will move out of the monitored water space during the network operation.

**Figure 8:**
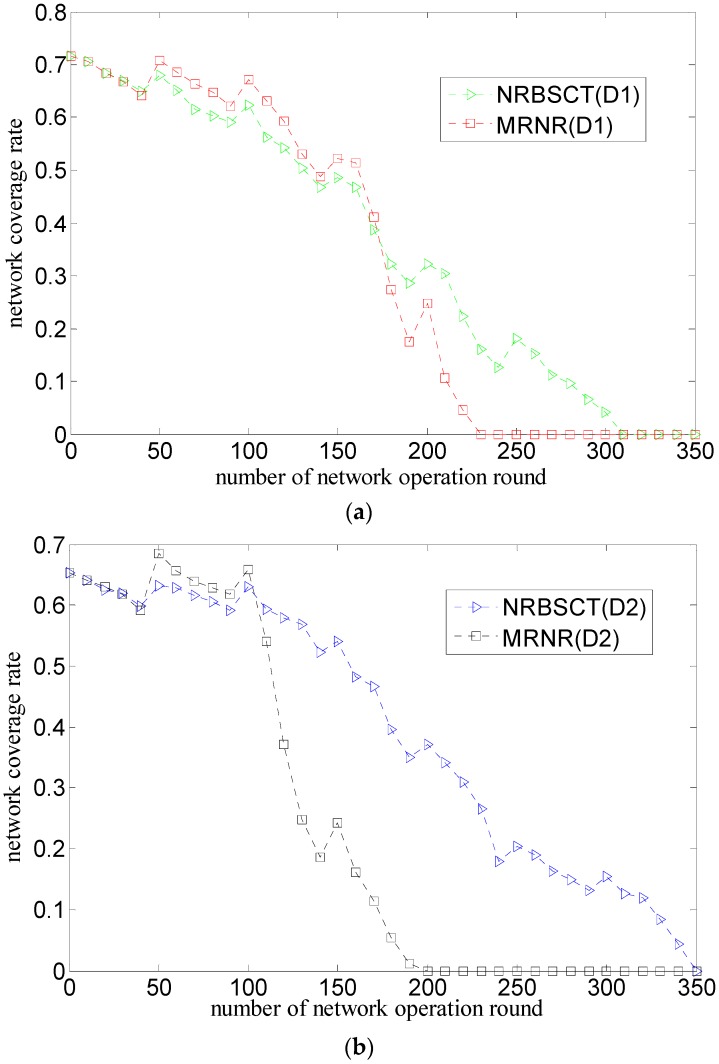

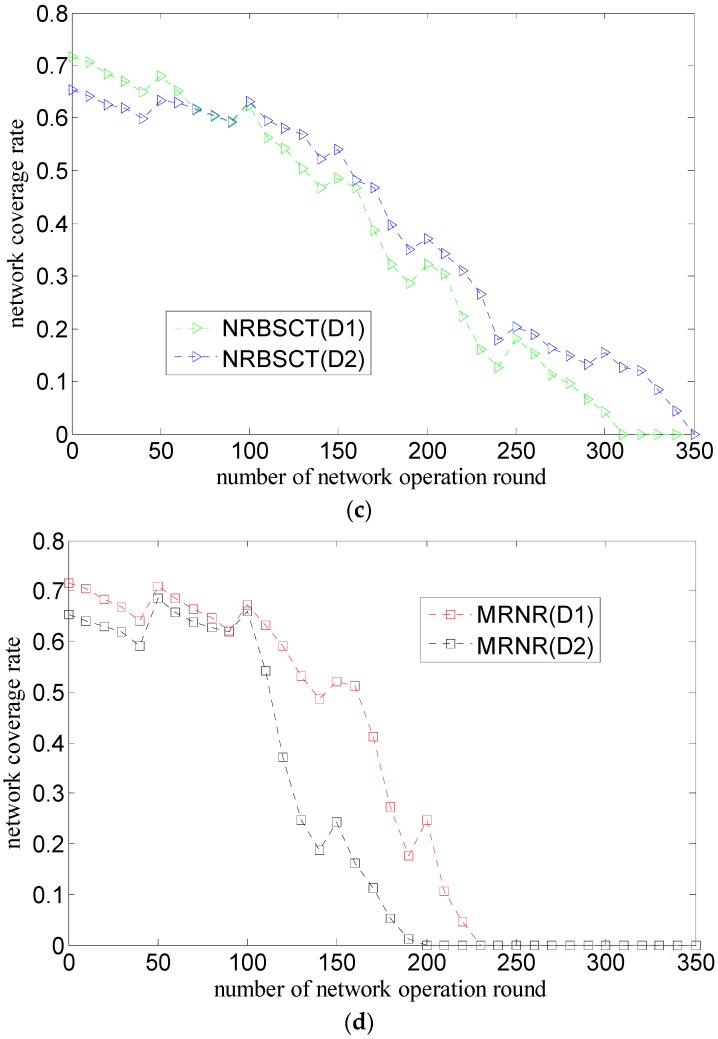
Comparison of network coverage rate during network operation: (**a**) different algorithms and same network distribution (D1); (**b**) different algorithms and same network distribution (D2); (**c**) different network distribution and same algorithm (NRBSCT); and (**d**) different network distribution and same algorithm (MRNR).

Figure 8 shows the comparison of the relationship between the network coverage rate and the round of network operation considering both the initial network distribution (D1 or D2) and the network redeployment algorithm (MRNR or NRBSCT), where the number of nodes is 30.

As shown in Figure 8a,b, for both of these algorithms, the network coverage rates will decrease during network operation from the whole. The reason is that some nodes drift out of the monitored space and some nodes die from energy depletion. However, when the network operation time is just the network adjustment moment, both of these two algorithms can contribute to the network coverage rate improvement to some extent since they propose the corresponding methods. Specifically, at the beginning of network operation (i.e., from the start to the 176 rounds for the initial network distribution D1 and to the 103 rounds for the initial network distribution D2), the MRNR algorithm can always achieve a higher network coverage rate than the NRBSCT algorithm; however, at the end of the network operation, the network coverage rate for the MRNR algorithm degrades dramatically with a relatively short network lifetime (i.e., 215 rounds for the initial network distribution D1 and 174 rounds for the initial network distribution D2), and that for the NRBSCT algorithm can remain a certain level for a longer network lifetime (i.e., 275 rounds for the initial network distribution D1 and 326 for the initial network distribution D2). The reason is that the MRNR algorithm only focuses on maximizing the network coverage rate, which results in a higher network coverage rate; however, compared with the MRNR algorithm, the proposed NRBSCT algorithm not only considers how to keep nodes from drifting out of the monitored space, but also tries to shorten node movement distance when the sink node conducts centralized optimization on leaf node locations, which can help to save movement energy consumption and keep the network coverage rate at a certain level for a longer network lifetime.

As shown in Figure 8c,d, for the NRBSCT algorithm, compared with the initial network distribution D1, since the nodes in the initial network distribution D2 tends to be located in the area near the sink node, which results more coverage overlaps, the network coverage at the beginning of network operation (i.e., from the start to the 78 rounds) is relatively lower. However, the initial network distribution D2 also results in less moving-out nodes during network operation and less movement distance during the network redeployment, which helps a lot to save the node movement energy consumption, so more nodes will save their energy to contribute to higher network coverage rates and the network lifetime can be prolonged from 275 rounds in the case of initial network distribution D1 to 326 rounds in the case of initial network distribution D2. However, that regularity cannot hold for the MRNR algorithm, since compared with the initial network distribution D1, the initial network distribution D2 is exactly less uniform, which not only means that there exists more coverage overlaps in the initial network operation, but also causes that more nodes will be involved in the network redeployment process, moving from the dense area close to the sink node to the sparse area far away from the sink node. Because of the relatively large movement energy consumption, too much movement distance will result in large movement energy consumption, which makes nodes die faster and degrades the network coverage rate. For the above reasons, the network coverage for the MRNR algorithm in the case of initial network distribution D2 cannot exceed that in the case of initial network distribution D1.

**Figure 9:**
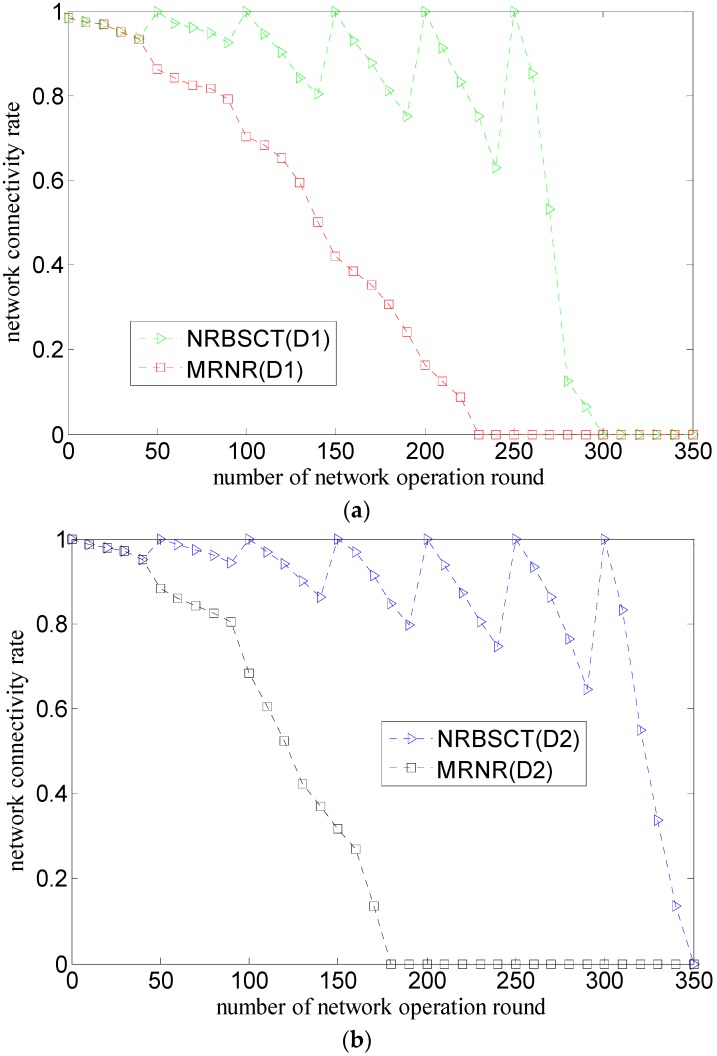

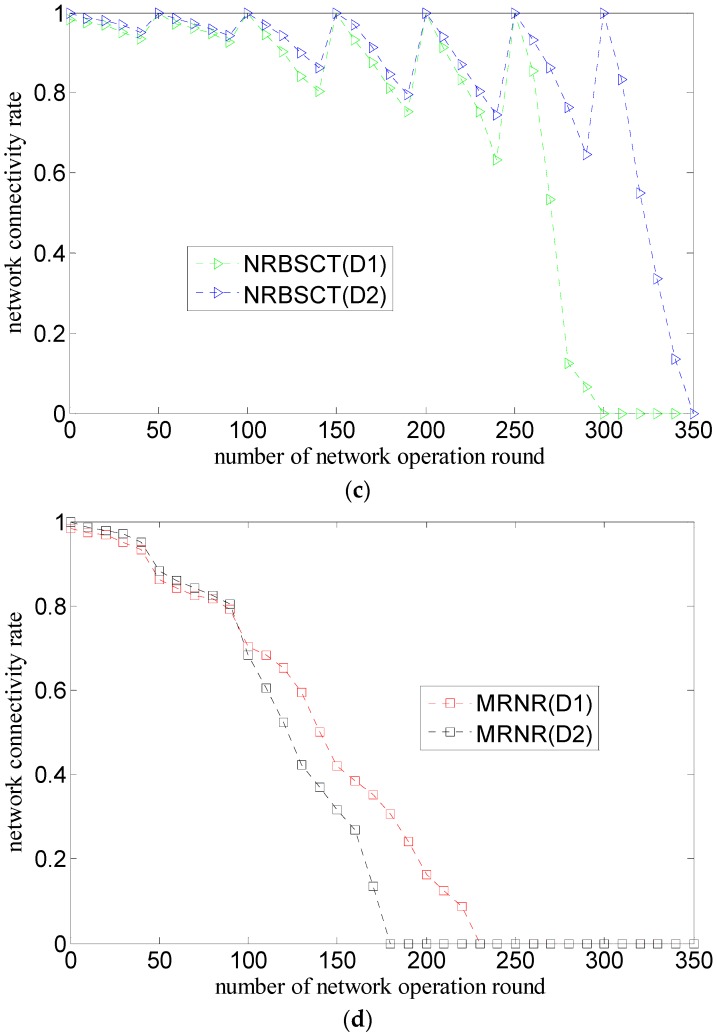
Comparison of network connectivity rate during network operation: (**a**) different algorithms and same network distribution (D1); (**b**) different algorithms and same network distribution (D2); (**c**) different network distribution and same algorithm (NRBSCT); and (**d**) different network distribution and same algorithm (MRNR).

Figure 9 shows the comparison of the relationship between the network connectivity rate and the round of network operation considering both the initial network distribution (D1 or D2) and the network redeployment algorithm (MRNR or NRBSCT), where the number of nodes is 30.

As shown in Figure 9a,b, for the MRNR algorithm, the network connectivity rate will decrease during network operation from the beginning to the network lifetime. The reason is that on the one hand, some nodes drift out of the monitored space and some nodes die from energy depletion; on the other hand, the MRNR algorithm only focuses on maximizing the network coverage rate and ignores how to slow the network connectivity rate decrease. However, for the NRBSCT algorithm, the network connectivity exhibits some fluctuations during its decrease. The reason for this is actually the consideration of slowing the network connectivity rate decrease. Specifically speaking, if the network operation time is not the network adjustment moment *T_ad_*, the network connectivity exhibits the decrease because of the node drift caused by water environment and node death caused by energy depletion. While, if the network operation time is just the network adjustment moment *T_ad_* (such as 50 rounds, 100 rounds, 150 rounds), the NRBSCT algorithm can firstly establish a stratified connected tree that takes the sink node as the root node, and then ensure that in the centralized optimization, the final destination location of node *s_i_* will not be *R_t_* away from the backbone nodes, which achieves full network connectivity and makes the value of the network connectivity bigger than that of the former or latter network operation time (i.e., the network connectivity exhibits the fluctuation phenomenon).

As shown in Figure 9c,d, for the NRBSCT algorithm, compared with the initial network distribution D1, the network connectivity rate in the case of the initial network distribution D2 is relatively higher, which may be the result of the fact that the majority of nodes in the initial network distribution D2 tend to be located in the area near the sink node. However, the same regularity can only hold for a short network operation time for the MRNR algorithm (i.e., 98 rounds), the reason is that at the network adjustment moments, in order to increase the network coverage rate, the MRNR algorithm requires nodes in the dense area near the sink node to move to the sparse area far away from the sink node, which will degrade the network connectivity.

**Figure 10:**
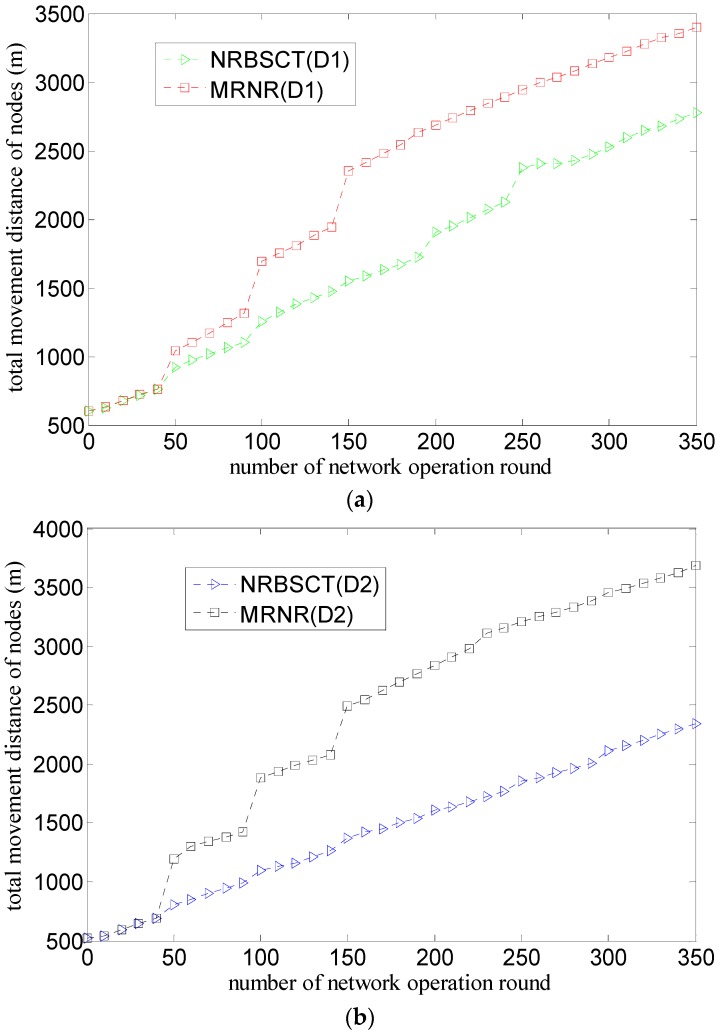

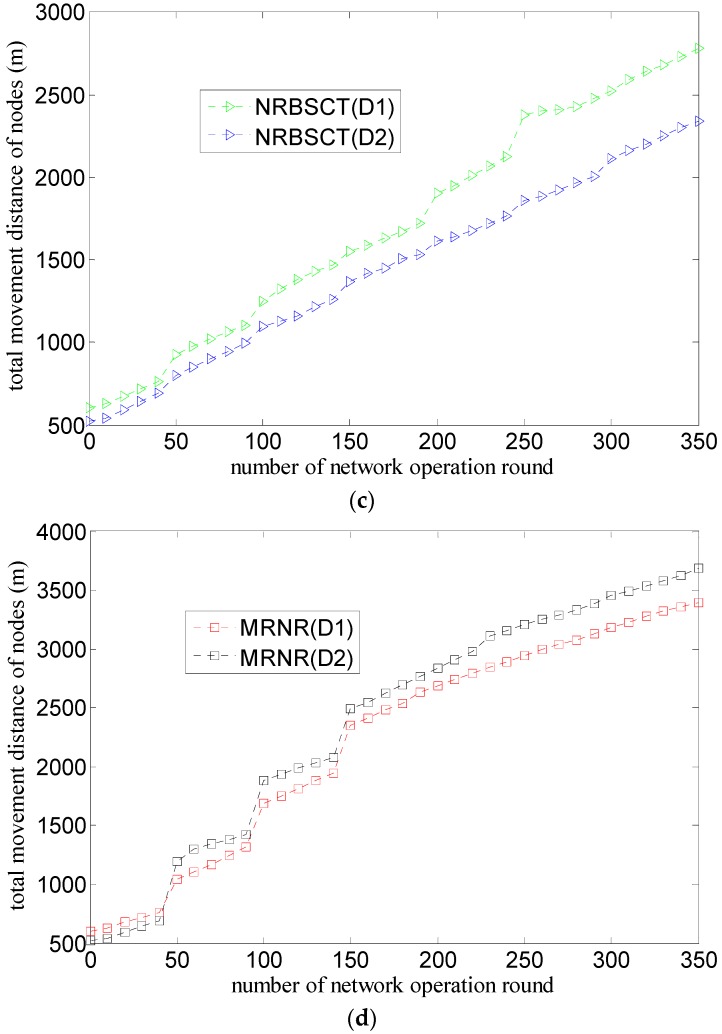
Comparison of total movement distance of nodes during network operation: (**a**) different algorithms and same network distribution (D1); (**b**) different algorithms and same network distribution (D2); (**c**) different network distribution and same algorithm (NRBSCT); (**d**) different network distribution and same algorithm (MRNR).

Figure 10 shows the comparison of the relationship between the total movement distance of nodes in the network and the round of network operation considering both the initial network distribution (D1 or D2) and the network redeployment algorithm (MRNR or NRBSCT), where the number of nodes is 30.

As shown in Figure 10a,b, the total movement distance of nodes will increase during network operation from the whole for both algorithms. The reason is that, on the one hand, nodes may drift with water environment; on the other hand, both redeployment algorithms require some nodes to move toward better locations to improve network monitoring quality when the network operation time is just the network adjustment moment. Compared with the former, the latter will cause larger movement distance, which can be demonstrated with the higher curve slopes at the network adjustment moments. However, the total movement distance for the NRBSCT algorithm is smaller than that of the MRNR algorithm, the reason is that compared with the latter, the former considers how to decrease the node movement distance from the left and consumed energy perspectives when the sink node conducts centralized optimization on the locations of leaf nodes, which avoids the large-scale node movement that may occur in the latter.

As shown in Figure 10c,d, for the NRBSCT algorithm, compared with the initial network distribution D1, the total movement distance of nodes in the network in the case of the initial network distribution D2 is relatively smaller, and there are two main reasons accounting for this phenomenon. Firstly, the movement of nodes in the formation of the initial network distribution D2 is comparatively smaller than that of the initial network distribution D1, since the majority of all the nodes only need a relatively small depth adjustment. Secondly, in the case of the initial network distribution D2, based on the fact that the majority of all the nodes are located in the area near the sink node and the minority of all the nodes are located in the area far away from the sink node, both the number of node moving out of the monitored space and the node movement distance produced in process of the network redeployment at the network adjustment moments are relatively smaller. However, for the MRNR algorithm, the same regularity can only hold for a short network operation time (i.e., before the 50 rounds) owning to the first reason mentioned above. Once the network operation time comes to the network adjustment moment, large numbers of node in the dense area near the sink node will be required to move towards the sparse area far away from the sink node for the purpose of increasing the network coverage rate, which will obviously cause larger node movement distance increase than that in the case of the initial network distribution D1.

**Figure 11:**
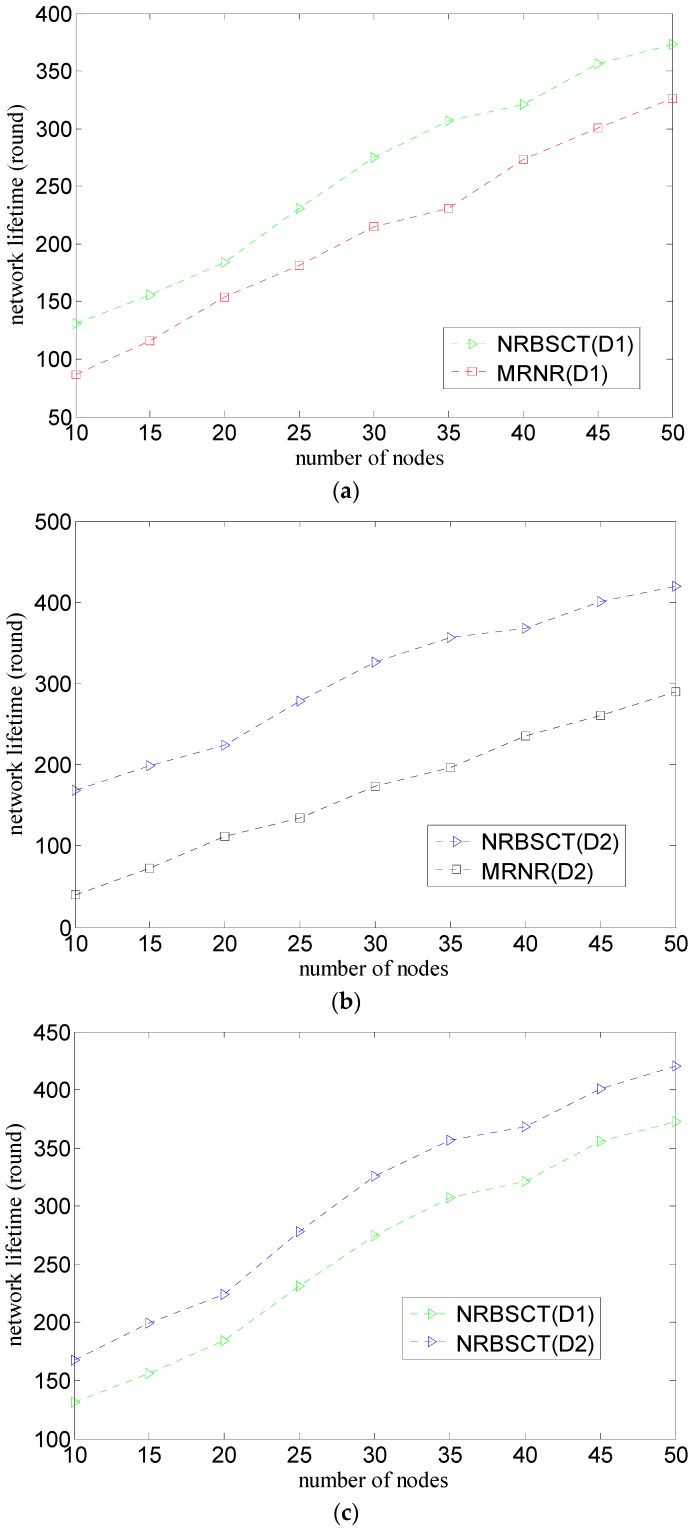

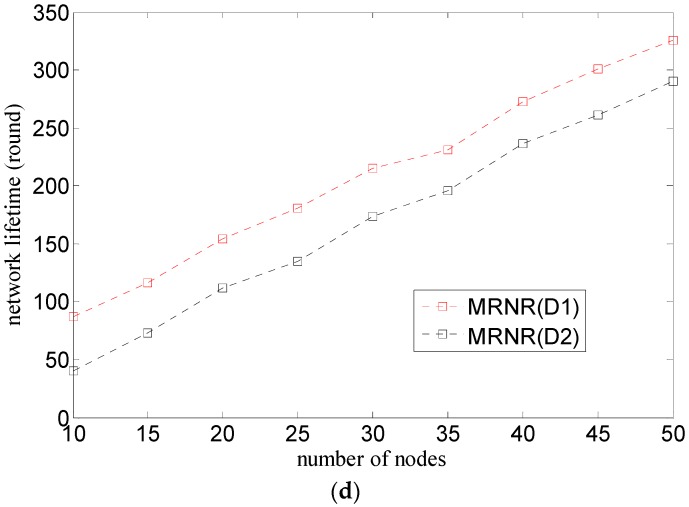
Comparison of network lifetime: (**a**) different algorithms and same network distribution (D1); (**b**) different algorithms and same network distribution (D2); (**c**) different network distribution and same algorithm (NRBSCT); and (**d**) different network distribution and same algorithm (MRNR).

Figure 11 shows the comparison of the relationship between the network lifetime and the number of nodes considering both the initial network distribution (D1 or D2) and the network redeployment algorithm (MRNR or NRBSCT), where the number of nodes varies from 10 to 50.

As shown in Figure 11a,b, compared with the MRNR algorithm, when the network scale is the same (i.e., the number of nodes is the same), the NRBST can always achieve a higher network lifetime. The reason is that when the sink node conducts centralized optimization, the MRNR algorithm only considers maximizing the network coverage rate, with node movement distance ignored; while the NRBSCT algorithm considers how to not only improve the network coverage and connectivity rates, but also decrease node movement distance (this is also shown in Figure 10). Since node movement energy consumption in water environment is much larger than other kinds of energy consumption, the node movement distance decrease will produce a significant network lifetime improvement.

As shown in Figure 11c,d, compared with the initial network distribution D1, when the network scale is the same (i.e., the number of nodes is the same), the network lifetime in the case of the initial network distribution D2 is relatively longer for the NRBST algorithm and is relatively shorter for the MRNR algorithm. This phenomenon also results from the facts that node movement energy consumption accounts for large percent of the total energy consumption of a node. Furthermore, it is easy to understand this phenomenon considering the total node movement distance shown in Figure 10.

## 5. Conclusions and Future Work

A NRBSCT algorithm is proposed because improving the network coverage and connectivity rates at the cost of the probably minimal node energy consumption to maintain excellent network monitoring quality as long as possible with full consideration of node drift with water environment is difficult for existing UWSNs node redeployment algorithms. At every network adjustment moment, nodes firstly perform the self-examination and adjustment on locations to keep themselves in the monitored space, which ensures that number of nodes in the monitored space can be as much as possible and that network monitoring quality can be as good as possible. Then, the network is stratified into a connected tree through broadcasting ready information level by level that takes the sink node as the root node, which helps to improve the network connectivity rate and even achieve full network connectivity. Last, the sink node conducts centralized optimization on locations of leaf nodes with synthetically considering the network coverage rate, network connectivity rate, and node movement distance, which is beneficial to lower the network connectivity decrease speed and improve the network lifetime. Since the centralized optimization mentioned above is actually a multi-objective optimal problem, which is hard to get the optimal solution since UWSNs are usually a large-scale, dynamical, and energy-limited system, how to get a near-optimal solution deserves our future research. Besides, since both the connected tree construction and centralized optimization performed by the sink node seem a little simple, how to find more sophisticated methods with better performances is obviously one of our future research directions. Although the models used in this paper are either similar with and improved from those used in the existing and compared MRNR algorithm (like the 3D random drift), or the supplements to the lacks of the necessary models (such as the models for communication energy consumption and movement energy consumption) in the existing and compared MRNR algorithm, they have the following limitations:

(1) The 3D random drift model adopted to simulate the node drift caused by the water environment is relatively simple, considering the complex flow changes for realistic conditions.
(2) In the description of the energy consumption calculation for transmitting information, the attenuation model of acoustic propagation is relatively rough considering the reasonable uncertainty bounds for realistic conditions.
(3) The movement energy consumption calculation method is also relatively idealized considering the complex current effects for realistic conditions.

Therefore, it is necessary to establish more practical models in the future. Moreover, although the algorithm simulation is conducted on the software platform, as a research team which has been keeping much focus on WSNs, we have also successfully developed some practical underwater nodes and water environment monitoring systems, which demonstrate that putting the proposed algorithm into the real application is one of our key research directions. If necessary, interested readers can contact us to learn about or improve the developing process of the real UWSNs designed by our team.

## Figures and Tables

**Table 1:** Differences between the related research works and NRBSCT algorithm.

Literature/Algorithm	Node Mobility	Consideration of Node Drift with Water Environment
[19,20]	Static	No
[21,22,27]	limited	Additional
[28]	limited	No
[23,25,29,30,31]	Free	No
[26]	Free	Additional
[32], NRBSCT	Free	Full

**Table 2:** Location changes of node *s_e_*.

Round	0	40	49	50 (after Adjustment)
Location	(115, 37, 16)	(123, 41, 19)	(125, 43, 21)	(115, 37, 16)
Inside/Outside	Inside	Outside	Outside	Inside

**Table 3:** Left energy description.

Node	*s*_2_	*s*_3_	*s*_8_	*s*_9_
Left energy (J)	75	61	77	57
Inside/Outside	Inside	Outside	Outside	Inside
Strong leaf node	Yes	No	Yes	No

**Table 4:** Main mathematical symbols.

Symbols	Meanings
*w*	cube side length
*d*	transmitting distance of information package
*M_b_*	size of information package
*T_p_*	transmitting time of information package
*S_v_*	transmission speed of information package
*f*	carrier acoustic signal frequency
*α(f)*	water absorption coefficient
*A(d)*	energy attenuation
*λ*	energy spreading factor
*E_tx_(d)*	energy consumption for transmitting information
*P_r_*	power threshold for receiving information package
*t_n_*	information package transmitting times for a node
*R_t_*	communication range
*C_e_*	communication energy consumption
*M_e_*	movement energy consumption
*m_d_*	movement distance
*m_u_*	energy consumption per movement distance
*P_e_*	controlling probability of node drift
*C_v_*	network coverage rate
*P_c_*	number of cube points covered
*P_t_*	total number of all cube points
*C_n_*	network connectivity rate
*n_c_*	number of nodes being able to communicate with sink node
*n*	number of all nodes
*t*	network operation time
*T_r_*	network adjustment cycle
*T_ad_*	network adjust moment
*E_i_*	energy of node *s_i_*
*E_d_*	energy threshold judging death of node
*E_y_*	energy threshold judging whether leaf node is strong enough
*L_f_*	network lifetime
*C_th_*	coverage rate threshold
*E_in_*	node initial energy
*R_s_*	sensing range
*T_m_*	node drift cycle
*M_r_*	ready information
*M_a_*	acknowledging information
*r_g_*	control coefficient

**Table 5:** Parameter settings.

Parameter Names	Parameter Values
Initial energy of node (*E_i_*)	500 J
Network coverage rate threshold (*C_th_*)	0.1
Energy consumption per movement distance (*m_u_*)	1.5 J/m
Size of information package (*M_b_*)	1 Kbit
Receiving Power threshold (*P_r_*)	0.05 w
Frequency of carrier acoustic signal (*f*)	25 kHz
Transmission speed of information package (*S_v_*)	5 kbps
Energy spreading factor (*λ*)	1.5
Sensing range of node (*R_s_*)	15 m
Communication range of node (*R_t_*)	25 m
Network adjustment cycle (*T_r_*)	50 round
Node drift cycle (*T_m_*)	5 round
